# Health literacy and the determinants of obesity: a population-based survey of sixth grade school children in Taiwan

**DOI:** 10.1186/s12889-016-2879-2

**Published:** 2016-03-22

**Authors:** Shu-Fang Shih, Chieh-Hsing Liu, Li-Ling Liao, Richard H. Osborne

**Affiliations:** Department of Health Promotion and Health Education, College of Education, National Taiwan Normal University, No. 162, Section 1, Heping East Road, Da-An District 106, Taipei, Taiwan; Department of Health Management, I-Shou University, No. 8, Yida Rd., Jiaosu Village, Yanchao District, Kaohsiung City, 82445 Taiwan; Deakin University Centre for Population Health Research, School of Health and Social Development, Geelong, Victoria Australia

**Keywords:** Children, Heath literacy, Health behavior, Body mass index, Taiwan

## Abstract

**Background:**

Health literacy has become an important health policy and health promotion agenda item in recent years. It had been seen as a means to reduce health disparities and a critical empowerment strategy to increase people’s control over their health. So far, most of health literacy studies mainly focus on adults with few studies investigating associations between child health literacy and health status. This study aimed to investigate the association between health literacy and body weight in Taiwan’s sixth grade school children.

**Methods:**

Using a population-based survey, 162,209 sixth grade (11–12 years old) school children were assessed. The response rate at school level was 83 %, with 70 % of all students completing the survey. The Taiwan child health literacy assessment tool was applied and information on sex, ethnicity, self-reported health, and health behaviors were also collected. BMI was used to classify the children as underweight, normal, overweight, or obese. A multinomial logit model with robust estimation was used to explore associations between health literacy and the body weight with an adjustment for covariates.

**Results:**

The sample consisted of 48.9 % girls, 3.8 % were indigenous and the mean BMI was 19.55 (SD = 3.93). About 6 % of children self-reported bad or very bad health. The mean child health literacy score was 24.03 (SD = 6.12, scale range from 0 to 32). The overall proportion of obese children was 15.2 %. Children in the highest health literacy quartile were less likely to be obese (12.4 %) compared with the lowest quartile (17.4 %). After controlling for gender, ethnicity, self-rated health, and health behaviors, children with higher health literacy were less likely to be obese (Relative Risk Ratio (RRR) = 0.94, *p* < 0.001) and underweight (RRR = 0.83, *p* < 0.001). Those who did not have regular physical activity, or had sugar-sweetened beverage intake (RRR > 1.10, *p* < 0.0001) were more likely to report being overweight or obese.

**Conclusions:**

This study demonstrates strong links between health literacy and obesity, even after adjusting for key potential confounders, and provides new insights into potential intervention points in school education for obesity prevention. Systematic approaches to integrating a health literacy curriculum into schools may mitigate the growing burden of disease due to obesity.

**Electronic supplementary material:**

The online version of this article (doi:10.1186/s12889-016-2879-2) contains supplementary material, which is available to authorized users.

## Background

Health literacy has become an important health policy and health promotion agenda item in recent years [1, 2]. It has been defined by the World Health Organization as “The characteristics and social resources needed for people to access, understand and use information to make decisions about health. Health literacy includes the capacity to communicate, assert and enact these decisions [3]”. The United Nations Economic and Social Council Ministers Declaration in 2009, highlighted health literacy as an important determinant of health and called for the development of actions to promote health literacy [2]. Furthermore, health literacy had been seen as a means to reduce health disparities and a critical empowerment strategy to increase people’s control over their health, their ability to seek information and their ability to take responsibility for their health [3, 4]. While policy initiatives exist in North America [5] and in the European Union, these have tended to focus only on adults. While there is much to gain through improving health literacy in adults, understanding earlier intervention opportunities in children is most certainly warranted. Improving health literacy in children may also increase health promoting behaviors, improve health service use and potentially increase health behaviors of the child’s family. Health literacy is a relatively new field and it is not clear whether it can be changed, however it should be possible to improve health knowledge, health choices and children’s and family's access to services thought effective interventions. Overall, it seems reasonable that strong health education programs should be able to improve health literacy and a range of future health outcomes.

A systematic review of 215 health literacy studies found that there were no validated tools to specifically measure health literacy in children younger than 12 years of age. While some tools had been applied in children or adolescents, they were adaptations of adult version [6]. In addition, the studies that focused on child health literacy used a definition and analyses that were highly medicalized. So far, only a few studies have considered child or adolescent health literacy in non-medical settings such as schools [7, 8]. While there is a substantial body of research on adults and/or clinical populations [9], the development of health literacy approaches in child and adolescent setting has been limited [10].

Most studies that have considered health literacy and children have focused on associations between parents or caregivers’ health literacy and health care of children [11–17] or health outcomes [18] with only a few exploring the role of children’s health literacy and their health outcomes. One study of children with Type 1 diabetes found that there was no relationship between children’s health literacy skill and hemoglobin A1C [19]. Another study found that child health literacy was negatively associated with BMI Z-scores in overweight children [20]. However, this relatively small study (*n* = 171) mainly focused on overweight children and used a health literacy instrument initially designed for adults. Given the dearth of research in this area, Keim-Malpass et al. [21] concluded that to advance this field, new approach to direct health literacy assessment of children or adolescents is warranted.

To advance the understanding of health literacy and health in children, the Taiwan Ministry of Education commissioned the development of a child-specific measure of health literacy within its national Health-Promoting Schools (HPS) initiative. A national survey was conducted among sixth grade school children and this provided the opportunity to gain a deeper understanding of health literacy to guide policy and programs in this setting. In Taiwan, the prevalence of overweight and obesity for elementary schools is 30.4 % according to the student examination data from the Ministry of Education [22]. Whether body weight is associated with health literacy is still unknown in Taiwan. The specific aim of the current study was to determine the association between health literacy and body weight using data from the Taiwan child health literacy test.

## Methods

### Participants

This research was sponsored, reviewed, and then conducted under the supervision of Taiwan’s Ministry of Education. The Ministry of Education approved the scientific protocol and ethical aspects of this study, and the use of the data collected. Since this was a policy research project initiated and overseen by the Government of Taiwan, specific Institutional Review Board approval was not required. Importantly, the conduct of the study was consistent with the ethical guidelines of the Helsinki Declaration for Ethical Principles for Medical Research Involving Human Subjects [23].

The Ministry of Health invited all schools to participate. An anonymous web-based National Survey of Child Health Literacy in Taiwan’s elementary schools was undertaken in April 2012. Participation was voluntary and a total of 2400 schools (83 %) took part. As this was part of a national approach to improve the quality of health education, written consent from individual students was not sought, however students and their parents were informed of the survey and it was voluntary. Completion of the survey was regarded as implied consent. There were 162,609 sixth grade (11–12 years old) children who completed the anonymous online survey with a response rate of 69.5 % at the school level. After excluding those with missing birth dates, there were 162,209 children in the analysis. The distribution of the gender in our sample is similar to data from the Ministry of Education. The percentage of gender for 6th grade boys and girls were about 52 and 48 % respectively [24].

### Measures

BMI was calculated using height, weight, age and gender using the extended International Body Mass Index Cut-offs proposed by the International Obesity Task Force (IOTF) and modified for Asian body types. Four groups were generated by combining underweight grade 1 to 3 (BMI < 16, 16 < =BMI < 17, and 17 < =BMI < 18.5), normal weight (18.5 < =BMI < =23), overweight (23 < BMI < =25, unofficial Asian cut-off) and overweight (25 < BMI < =27), obesity (27 < BMI < =30, unofficial Asian cut-off), obesity (30 < BMI < =35), and morbid obesity (35 < BMI). While height and weight were self-reported by the students, teachers helped students to answer this question. In Taiwan, weights and heights were measured every semester and the teachers had the data for all students. Therefore, the data are likely to be more reliable than most large scale surveys of obesity.

The Taiwan Child Health Literacy test is a course-based test. In order to ensure the test results were relevant to educators, we designed the test based on the learning taxonomy proposed by Bloom [25], including the following domains: cognitive, psychomotor, and affective domain. Therefore, in the cognitive domain, the curriculum sought to equip students with adequate knowledge in certain health conditions. For the psychomotor domain, skills for being able to take actions in certain scenarios was sought, and for the affective domain, the curriculum sought to equip students with positive values in certain health conditions, or have healthier decision intentions.

The Taiwan Child Health Literacy test was developed with sixth grade students in elementary schools using a grounded approach capturing health literacy elements across the 10 health education areas of the Taiwan National Health Education Curriculum. The original test includes 40 multiple choice questions to elicit knowledge, attitudes and preferred behavior in response to a vignette (short narrative or story of three to five sentences) linked to one or more of the health education curriculum areas. The test was developed through a series of focus groups, expert review and then field testing in 273 students from 8 schools. The final test consisted of 32 questions with item discrimination of 0.55 to 1.89 and item difficulty of −1.7 to 0.41 according to Item Response Theory. The Cronbach reliability is 0.87. The questionnaire has been reported elsewhere [26], and an example question is shown in Additional file 1 and is available from the corresponding author.

Information was also collected on sex, ethnicity, self-rated health, exercise for at least 30 min every day, and consumption of sugar-sweetened beverages (SSB). The behaviors were categorized into: (1) exercise at least 30 min every day and had no SSB intake; (2) either exercise at least 30 min every day or had no SSB intake; (3) no exercise at least 30 min every day and SSB intake. Self-rated health was measured using single item, with responses scored as very good, good, fair, bad or very bad.

The data were retained by the Ministry of Education, and those who are interested in using the data should submit an application to the Ministry.

### Statistical analysis

Descriptive statistics included frequencies and percentages of research variables. Chi-square test was used to test the bivariate associations between category of BMI and health literacy, as well as other covariates such as sex, ethnicity, self-rated health status, and health behaviors. Since the dependent variable was four categories of BMI (underweight, normal, overweight, and obese), a multinomial logistic regression model was used to analyze the factors associated with the probability of being underweight or overweight, or obesity relative to a normal weight with an adjustment for covariates. Since students were nested within schools, however, data on these clusters were not available; we used the Huber Sandwich Estimator to provide robust standard errors. *P* values of less than 0.05 for associations were considered to indicate statistical significance. Stata 12 software was used for all analyses.

## Results

### Participant characteristics

Table 1 shows the characteristics of 162,209 grade six school children across 2400 elementary schools in Taiwan. Of these children, more than half were girls, and less than 4 % were from indigenous groups. However, around 32 % of children were not certain about their ethnicity. Self-rated health was rated as bad or very bad by 6 %. Only around 40 % reported regular physical activity and had no SSB intake, and almost 50 % of children had at least one healthy behavior, and about 11 % of children had no regular physical activity but had SSB intake. According to IOTF cutoffs, less than 50 % of children had normal weight, around 11 % were underweight, more than 25 % were overweight, and more than 15 % were classified as obese. The mean BMI was 19.55 % (SD = 3.93) and the mean of child health literacy score was 24.03 (SD = 6.12, scale range from 0 to 32).

**Table 1:** Characteristics of study sample (*n* = 162,209)

Characteristics	n	%
Sex		
Girl	79,250	48.9
Boy	82,959	51.1
Ethnicity		
Hakka, Taiwanese, and Chinese	101,575	62.6
Indigenous	6,118	3.8
Immigrants	3,159	2.0
Others/Unknown/Uncertain	51,357	31.7
Health literacy (quartiles)		
Lowest 25 % (0–21)	43,062	26.6
25^th^ to 50^th^ percentile (22–26)	47,848	29.5
50^th^ to 75^th^ percentile (27–29)	44,874	27.7
Highest 25 % (30–32)	26,425	16.3
Self-rated health status		
Bad or very bad	9,736	6.0
Normal	62,069	38.3
Good	45,747	28.2
Very Good	44,657	27.5
Healthy behaviors (Regular physical activity, Sugar-sweetened beverage (SSB) intake)		
Regular physical activity and no SSB intake	64,034	39.5
Had only one of the healthy behaviors (either regular physical activity or no SSB intake)	80,500	49.6
Had no regular physical activity and consumed SSB	17,675	10.9
BMI		
Normal	78,101	48.2
Underweight	18,015	11.1
Overweight	41,423	25.5
Obese	24,670	15.2

### Distribution of BMI across demographic characteristics

Table 2 illustrates the distribution of BMI across different individual characteristics. Among girls, over half had normal weight (53.25 %) while 23.92 % were classified as overweight, 13.00 % were underweight, and 9.83 % were classified as obese. 43.27 % of boys had normal weight, 27.08 % were classified as overweight, 20.35 % were classified as obese; and 9.30 % were classified as underweight. 44.97 % of the indigenous children had normal weight, 29.05 % were classified as overweight, and 18.36 % were classified as obese. All covariates were statistically associated with categories of BMI.

**Table 2:** Distribution of BMI across different characteristics

	BMI								
Characteristics	Normal		Underweight		Overweight		Obese		*P*-value^1^
	n	%	n	%	n	%	n	%	
Health literacy (quartiles)									<0.0001
Lowest 25 % (0–21)	19,580	45.47	4,954	11.50	11,051	25.66	7,477	17.36	
25^th^ to 50^th^ percentile (22–26)	22,971	48.01	5,276	11.03	12,110	25.31	7,491	15.66	
50^th^ to 75^th^ percentile (27–29)	22,190	49.45	4,847	10.80	11,403	25.41	6,434	14.34	
Highest 25 % (30–32)	13,360	50.56	2,938	11.12	6,859	25.96	3,268	12.37	
Sex									<0.0001
Girl	42,204	53.25	10,299	13.00	18,957	23.92	7,790	9.83	
Boy	35,897	43.27	7,716	9.30	22,466	27.08	16,880	20.35	
Ethnicity									<0.0001
Hakka, Taiwanese, and Chinese	49,357	48.59	11,156	10.98	25,761	25.36	15,301	15.06	
Indigenous	2751	44.97	467	7.63	1,777	29.05	1,123	18.36	
Immigrants	1440	45.58	364	11.52	816	25.83	539	17.06	
Others/Unknown/Uncertain	24,553	47.81	6,028	11.74	13,069	25.45	7,707	15.01	
Self-rated health status									<0.0001
Bad or very bad	2351	24.15	1,127	11.58	2,486	25.53	3,772	38.74	
Normal	27,917	44.98	7,028	11.32	16,493	26.57	10,631	17.13	
Good	24,839	54.30	4,901	10.71	11,162	24.40	4,845	10.59	
Very Good	22,994	51.49	4,959	11.10	11,282	25.26	5,422	12.14	
Healthy behaviors (Regular physical activity, Sugar-sweetened beverage (SSB) intake)									<0.0001
Regular physical activity and no SSB intake	32,432	50.65	6,988	10.91	16,050	25.06	8,564	13.37	
Had only one of the healthy behaviors (either regular physical activity or no SSB intake)	37,591	46.70	9,080	11.28	20,867	25.92	12,962	16.10	
Had no regular physical activity and consumed SSB	8078	45.70	1,947	11.02	4,506	25.49	3,144	17.79	

^1^ Chi-square test showing bivariate association between BMI category and other variables

Among children with a health literacy score in the bottom quartile, 43.02 % were classified as overweight or obese. For those children who perceived that they had bad or very bad health status, 38.74 % were classified as obese. This percentage was much higher when compared to the percentage of obese children who reported their health as normal (17.13 %), or good (10.59 %), or very good (12.14 %). Finally, obese children were less likely to report both regular activity and no SSB (13.37 %) compared with those with one poor health habit (16.10 %) or two poor health habits (17.79 %).

### Association between BMI and health literacy

After controlling for sex, ethnicity, self-rated health status and health behaviors, being in the highest quartile of heath literacy was associated with lower likelihood of being either underweight or obese. However, higher health literacy was associated with higher likelihood of being overweight than having normal weight (Table 3). When compared to those with lowest health literacy, those who had higher health literacy (i.e., those below 25th compared with above 25th percentile) were less likely to be underweight than to be in the normal group. Compared with those who had two healthy behaviors, those who had only one healthy behavior were more likely to be underweight than to have normal weight (Relative Risk Ratio (RRR) = 1.05, *p* < 0.001). Those who did not have regular physical activity or consumed SSB (RRR = 1.12, *p* < 0.001), or those who did not have regular physical activity and consumed SSB (RRR = 1.10 *p* < 0.001) were more likely to be overweight than to have normal weight. Similarly, Those who did not have regular physical activity or consumed SSB (RRR = 1.27, *p* < 0.001) or those who did not have regular physical activity and consumed SSB (RRR = 1.31, *p* < 0.001) were more likely to be obese than to have normal weight. In terms of ethnicity, compared with Hakka, Taiwanese and Chinese groups, indigenous groups were less likely to be underweight than to have normal weight (RRR = 0.73, *p* < 0.001), and this group was much more likely to be overweight (RRR = 1.24, *p* < 0.001) or obese (RRR = 1.32, *p* < 0.001) than to have normal weight.

**Table 3:** Multinomial logistic regression model of the association between BMI and health literacy

Characteristics	Relative Risk Ratio	95 % CI	Robust Std. Err.	z	P > |z|
Underweight Group (Ref: Normal Group)					
Sex (Ref: Girl)					
Boy	0.87	(0.84–0.90)	0.02	−7.77	<0.001
Ethnicity (Ref: Hakka, Taiwanese, and Chinese)					
Indigenous	0.73	(0.66–0.81)	0.04	−6.06	<0.001
Immigrants	1.12	(1.00–1.26)	0.07	1.90	0.06
Others/Unknown/Uncertain	1.07	(1.03–1.11)	0.02	3.81	<0.001
Health literacy (quartile) (Ref: Lowest 20 %)					
25^th^ to 50^th^ percentile (22–26)	0.88	(0.85–0.92)	0.02	−5.53	<0.001
50^th^ to 75^th^ percentile (27-29)	0.83	(0.79–0.87)	0.02	−7.98	<0.001
Highest 25 % (30–32)	0.83	(0.79–0.87)	0.02	−6.96	<0.001
Self-rated health status (Ref: Bad or very bad)					
Normal	0.53	(0.49–0.58)	0.02	−16.23	<0.001
Good	0.43	(0.39–0.46)	0.02	−21.41	<0.001
Very Good	0.46	(0.43–0.50)	0.02	−19.11	<0.001
Health behaviors (Ref: Regular physical activity and no SSB intake)					
Had only one of the healthy behaviors (either regular physical activity or no SSB intake)	1.05	(1.01–1.09)	0.02	2.75	0.01
Had no regular physical activity and consumed SSB	0.99	(0.93–1.05)	0.03	−0.33	0.74
Overweight Group (Ref: Normal Group)					
Sex (Ref: Girl)					
Boy	1.49	(1.45–1.53)	0.02	30.73	<0.001
Ethnicity (Ref: Hakka, Taiwanese, and Chinese)					
Indigenous	1.24	(1.17–1.32)	0.04	6.88	<0.001
Immigrants	1.03	(0.94–1.12)	0.05	0.65	0.51
Others/Unknown/Uncertain	0.98	(0.95–1.00)	0.01	−1.69	0.09
Health literacy (quartile) (Ref: Lowest 25 %)					
25^th^ to 50^th^ percentile (22–26)	1.01	(0.98–1.04)	0.02	0.50	0.62
50^th^ to 75^th^ percentile (27–29)	1.03	(0.99–1.06)	0.02	1.59	0.11
Highest 25 % (30–32)	1.07	(1.02–1.11)	0.02	3.16	<0.001
Self-rated health status (Ref: Bad or very bad)					
Normal	0.55	(0.52–0.58)	0.02	−19.66	<0.001
Good	0.41	(0.39–0.44)	0.01	−28.45	<0.001
Very Good	0.43	(0.41–0.46)	0.01	−26.63	<0.001
Health behaviors (Ref: Regular physical activity and no SSB intake)					
Had only one of the healthy behaviors (either regular physical activity or no SSB intake)	1.12	(1.09–1.15)	0.01	8.41	<0.001
Had no regular physical activity and consumed SSB	1.10	(1.05–1.15)	0.02	4.33	<0.001
Obese Group (Ref: Normal Group)					
Sex (Ref: Girl)					
Boy	3.02	(2.92–3.12)	0.05	65.71	<0.001
Ethnicity (Ref: Hakka, Taiwanese, and Chinese)					
Indigenous	1.32	(1.22–1.42)	0.05	7.06	<0.001
Immigrants	1.03	(0.93–1.15)	0.06	0.64	0.52
Others/Unknown/Uncertain	0.89	(0.87–0.92)	0.01	−6.65	<0.001
Health literacy (quartile) (Ref: Lowest 25 %)					
25^th^ to 50^th^ percentile (22–26)	1.03	(0.99–1.07)	0.02	1.31	0.19
50^th^ to 75^th^ percentile (27–29)	1.01	(0.97–1.06)	0.02	0.67	0.50
Highest 25 % (30–32)	0.94	(0.89–0.99)	0.02	−2.52	0.01
Self-rated health status (Ref: Bad or very bad)					
Normal	0.22	(0.21–0.24)	0.01	−50.13	<0.001
Good	0.11	(0.10–0.12)	0.00	−68.85	<0.001
Very Good	0.12	(0.11–0.13)	0.00	−66.38	<0.001
Health behaviors (Ref: Regular physical activity and no SSB intake)					
Had only one of the healthy behaviors (either regular physical activity or no SSB intake)	1.27	(1.23–1.31)	0.02	14.11	<0.001
Had no regular physical activity and consumed SSB	1.31	(1.24–1.38)	0.03	10.20	<0.001

## Discussion

This is the first study to systematically investigate the association between child health literacy and body weight in school children. The use of a specific child health literacy instrument in a large population - based sample has shown that health literacy is an independent predictor of BMI. Importantly, those with the highest health literacy are those less likely to be obese after adjustment for the usual demographic predictors of BMI.

Our findings are similar to a much smaller study undertaken by Sharif et al. [20], who examined functional health literacy (reading and numeracy-related health literacy) in obese children however, our study provides clearer evidence given our health literacy test was children specific and nationally representative. According to Taiwan’s Ministry of Education, the percentage of overweight and obese elementary school children in Taiwan is 30.4 % [22]. Childhood obesity is an important predictor of adult obesity and appears to increase the risk of subsequent morbidity, whether or not obesity persists into adulthood. Conversely, underweight is also a public health problem that needs to be addressed in Taiwan. Therefore, weight management has become one of the most important health agenda in Taiwan Unfortunately it is mainly considered from the obesity management perspective without a substantive focus on underweight. We found that underweight is associated with low health literacy and this provides guidance for future interventions. Sharif et al. [20] pointed out that in recent decades, when dealing with child health issues, we not only tend to ignore the improvement of self-management skills, but also provide little emphasis on the effects that heath literacy has on health outcomes. Systematic approaches to providing adequately nutritional education, dietary intervention, encouragement of regular physical activity, as well as changing environments and food supply, are needed to prevent obesity and to reduce the increase of obesity-related disorders among children [27]. However, in order to enable children to participate in health management in the long term, as well as being able to avoid the temptations of junk foods such as sugar beverage in their daily environments, we need to improve children’s health literacy and nurture our children to “have knowledge, personal skill, and confidence to take action and enhance individual’s and community’s health by changing individual’s lifestyle and living conditions [28]”.

In our study, we did find that those with higher health literacy were less likely to be underweight than to have normal weight. The potential reasons for this are unclear. It may be due to children with low health literacy lived in regions or families with very poor access to food, or they were affected by an eating disorder or other condition. On the other hand, we found that children with lower health literacy were more likely to report being obese. While the study controlled for key potential confounders, it is likely that a parental health literacy effect is present, i.e., children living in more affluent and well-educated family environments, may have greater access to excess high calorie foods. Previous research has indicated that an association may exist between limited caregiver literacy skills and indicators of poor child nutrition [6].

This study has several policy implications. First, given the association between a child’s health literacy and their body weight, health literacy may offer a novel approach for tackling obesity problems through schools. While causal conclusions cannot be assumed in this cross-sectional study, systemic health education programs aimed at improving health literacy are likely to directly impact on obesity rates. It is likely that school-based interventions to improve health literacy will stimulate casual pathways related to physical activity and SSB intake that would in turn generate positive behavior patterns in children before adulthood.

This study had some limitations. First, due to anonymous data collection procedure, we are not able to identify the location of the schools. Second, while the sample size is large and a great majority of schools participated (83 %) it is possible that children from less affluent families did not participate, therefore introducing a systematic bias. While the direction of the bias is unknown, it is possible that our results are an underestimate of the association between health literacy and body weight in children. Due to the web-based and anonymous design of our survey, it is impossible to compare the respondents and non-respondents. However, there are no substantial differences between the non-participating schools and participating schools. It is likely that the complete picture of health literacy challenges of Taiwanese children is somewhat worse than what we report. This is because the missing cases are more likely to include those with the poorest general literacy and come from low socioeconomic family backgrounds. Given this, the health literacy estimates in the present study are likely to be conservative. The research may be improved in the future through face to face interviews and the collection of information on children’s and parent’s readiness to change or self-efficacy, as well as parental health literacy, demographics and socioeconomic status. In addition, almost a third of children did not answer the ethnicity question due to lack of awareness of their origins. This leaves a gap in our understanding of whether specific ethnic groups have greater health literacy needs and how this might affect obesity prevalence in Taiwan.

## Conclusions

This study provides some directions for future research and policy. A longitudinal study is needed alongside a child health surveillance system. We hypothesize that the quality of health education determines health literacy, which in turn, influences health behavior and health risk factors such as eating habits, physical activity, and other factors such as propensity to adopt healthy behaviors. It is possible that schools with strong health education programs may develop children with high health literacy and induce upward pressure in families and thus be a mechanism to reduce family and community obesity. We suggest that governments might consider applying routine child health literacy tests and monitoring the development of health literacy in individuals where health education, health behaviors and health and social outcomes are tracked throughout the school years. As the obesity epidemic continues to rise in many parts of the world, new surveillance systems that monitor the impact of education and social policy are needed. The use of health literacy surveys may be a much needed approach to identifying novel intervention points to reduce obesity and underweight.

## Additional file

Additional file 1: Health Report (Personal hygiene). (DOC 230 kb)
