# Supplementary material for: Health literacy and the determinants of obesity: a population-based survey of sixth grade school children in Taiwan
Source: BMC Public Health. 2016 Mar 22;16:280. doi: 10.1186/s12889-016-2879-2 (PMC4802836; doi:10.1186/s12889-016-2879-2)
Supplement: Additional file 1: — Health Report (Personal hygiene). (DOC 230 kb) [file 12889_2016_2879_MOESM1_ESM.doc]

Additional File 1


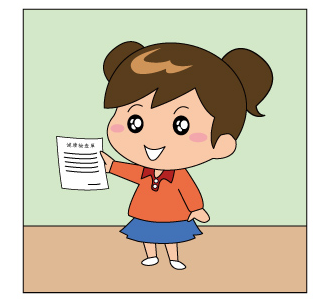
**Health Report (Personal hygiene)**

Every student received their health report today. I did not understand the results on my report until I consulted the school nurse. Then, I understood that I should start to improve my healthy status.

**Q1: Which description about health examination do you think is the most correct?**

1. Everyone should have an oral examination twice a year even one has no tooth decay.
2. People should control their body weight once their BMI has risen to 19.5, which means obesity.
3. If someone’s uncorrected vision is 0.5 on their left eye, one should go to the eyeglasses store immediately to get a pair of glasses for their near-sightedness.
4. The one, whose uncorrected vision in one eye is normal, and then one doesn’t have to correct the other eye for near-sightedness.


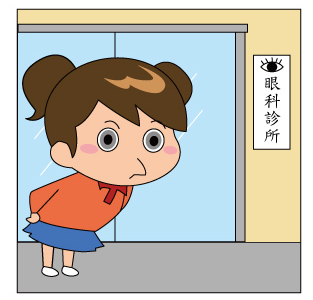


Then, my parents took me to the ophthalmic hospital, and the ophthalmologist diagnosed that I had pseudo myopia. My eyesight won’t recover unless I undergo a period of treatment.

**Q2: Which behavior would you use to protect your eyesight?**

1. I will relax my eyes when I watch something every 30 minutes.
2. I will borrow my friends’ glasses to ease my eyes when I can’t see clearly.
3. I will buy eye-drops to protect my eyes.
4. I will narrow my eyes as much as possible to lessen the danger of injury to my eyes.


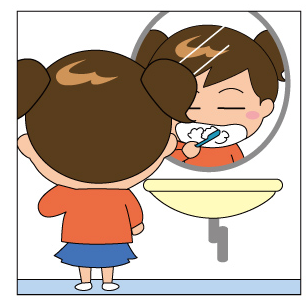


Speaking of oral health, I not only have a very good habit of brushing my teeth but use I use the Bass brushing technique, so I can keep my teeth healthy.

**Q3: Which one is the most important time for brushing to prevent tooth decay?**

1. Brush teeth after eating.
2. Brush teeth once after waking up.
3. Brush teeth before eating.
4. Brush teeth before going to bed.

**Q4: What would you like to do to take care of your teeth?**

1. Brush teeth the correct way after eating.
2. Eat lesser to prevent food particles getting stuck between my teeth.
3. Chew sugarless gum after eating.
4. Regularly use a mouthwash.
